# Fifteen-year Single Center Experience with the “Giessen Hybrid” Approach for Hypoplastic Left Heart and Variants: Current Strategies and Outcomes

**DOI:** 10.1007/s00246-014-1015-2

**Published:** 2014-09-02

**Authors:** Dietmar Schranz, Anna Bauer, Bettina Reich, Blanka Steinbrenner, Sabine Recla, Dorle Schmidt, Christian Apitz, Josef Thul, Klaus Valeske, Jürgen Bauer, Matthias Müller, Christian Jux, Ina Michel-Behnke, Hakan Akintürk

**Affiliations:** Pediatric Heart Center, Justus-Liebig University, Feulgenstr. 12, 30385 Giessen, Germany

**Keywords:** Hypoplastic left heart syndrome, Hypoplastic left heart complex, Hybrid approach

## Abstract

Presented is a retrospective outcome study of a 15-year single institutional experience with a contemporary cohort of patients with hypoplastic left heart syndrome and complex that underwent a “Giessen Hybrid” stage I as initial palliation. Hybrid approach consisting of surgical bilateral pulmonary artery banding and percutaneous duct stenting with or without atrial septum manipulation was developed from a rescue approach to a first-line procedure. Comprehensive Aristotle score defined pre-operative condition. Fifteen-year follow-up mortality is reported as occurring within the staged univentricular palliation or before and after biventricular repair. Hybrid stage I was performed in 154 patients; 107 should be treated by single ventricle palliation, 33 by biventricular repair (BVR), 7 received heart transplantation, and 7 were treated by comfort care, respectively. Overall 34 children died. The Aristotle score (mean value 18.2 ± 3) classified for univentricular circulations in newborns did not have statistical impact on the outcome. Two patients died during stage I (1.2 %), and the interstage I mortality was 6.7 %, and stage II mortality 9 %, respectively. Stage III was up to now performed in 57 patients without mortality. At 1 year, the overall unadjusted survival of HLHS and variants was 84 % and following BVR 89 %, respectively. The Fifteen-year survival rate for HLHS and variants was 77 %, with no significant impact of birth weight of less than 2.5 kg. In conclusion, Hybrid stage I fulfilled the criteria of life-saving approach. In our institution, Hybrid procedure replaced Norwood-staged palliation with a considerable mid- and long-term survival rate. Considering interstage mortality close surveillance is mandatory.

## Introduction

Hybrid approach expands the surgical options for patients born with hypoplastic left heart syndrome (HLHS) and newborns with multiple left heart obstructions, summarized as hypoplastic left heart complex (HLHC) [18]. Despite significant progress, surgical outcome for high-risk patients with HLHS remains suboptimal [3, 8, 11, 17, 20, 22, 25]. The hybrid palliation lessens the initial operative risk [1] and is hypothesized to improve in particular neurological survival [16]; however, the outcome of this sequential approach is unknown. Improved operative outcomes have resulted in the increasing use of the surgical palliation in high-risk patients, but at the expense of considerable morbidity and mortality [5, 6]. The early success with the hybrid approach reported by Akintuerk et al. [1, 2] and Galantowicz et al. [9, 10] have prompted the increasing use of this strategy in order to minimize the deleterious impact of the conventional surgical intervention on high-risk patients [4, 8, 14, 26]. Moreover, aggravating factors as low birth weight and surgical complexity on survival supported the idea that a less extensive neonatal procedure could improve the outcome in these patients [11, 22]. Therefore, we present the overall single institutional 15-year experience with an unselected cohort of HLHS and HLHC patients who underwent a Hybrid procedure as initial palliation providing their mid- and long-term outcome.

## Patients and Methods

This is a longitudinal review of all patients with HLHS, or it’s variants as well as HLHC were admitted between June 1998 and June 2013 to the Pediatric Heart Center of the University Hospital Giessen, in whom a Hybrid stage I approach was performed. Patient characteristics and cardiac anatomy are illustrated in Tables 1 and 2. The patients are grouped according their neonatal appraisal to undergo univentricular palliation (HLHS-group A) and biventricular repair (HLHC-group B). In short, the definition of HLHC in this study involves following criteria: borderline/obstructive left heart structures, duct-dependent circulation of the lower body, and antegrade flow to the ascending aorta. The only exception to this definition is the diagnosis of aortic atresia with the presence of ventricular septal defect and two ventricles. A small group of patients (group C) received Hybrid stage I procedure for primary listing to orthotopic heart transplantation (HTX) or comfort care after hybrid stage I.

**Table 1:** Patient demographics of HLHS and Variants

Demographic Data	Value (*n* = 121)
Male sex, n (%)	80 (66.1 %)
Low Birth Weight (<2.5 kg)	18 (14.8 %)
Genetic abnormality	9 (7.4 %)
Primary cardiac anatomy	
HLHS subtype	
MA, AA	34 (28.1 %)
MS, AA	33 (27.3 %)
MA, AS	6 (5 %)
MS, AS	28 (23.1 %)
Variants of hypoplastic left heart syndrome	20 (16.5 %)
ccTGA+	5
AA	2
Ebstein	2
IAA	2
TAPVR	1
Unbalanced AVSD	4
DORV+	4
MA	2
TAPVR	1
TGA + IAA	1
DILV + TGA + IAA (hAOA) +MS	4
Other	3

**Table 2:** Patient demographics of HLHC

Demographic Data	Value (*n* = 33)
Male sex, n (%)	17 (52 %)
Low Birth Weight (<2.5 kg)	10 (30 %)
Genetic abnormality	10 (30 %)
Main primary cardiac anatomy	
IAA + VSD + hypoplastic AAO	9 (27 %)
Syndromale	7
DORV + dTGA + hypoplastic AAO	1
Shone—complex	7
Aortic stenosis + hypoplastic AAO	7
Others with HLHC	9
Main primary cardiac anatomy	
Miscellaneous intra-cardiac repairs	25
Norwood-Rastelli/Yasui-operation	4
Rastelli-operation	3
Ross-Konno + AOA-reconstruction	1

Retrospective data collection included variables at admission, operative and postoperative parameters, as well as follow-up information during a 15-year observational period. Risk adjustment was calculated for each patient of the HLHS and variants group A via comprehensive Aristotle score. However, considering that Aristotle comprehensive score is used to classify HLHS in context of a Norwood stage I procedure with a basis score of 14,5 and to adjust for the complexity of the individual patient by taking into account patient specific “procedure-independent” characteristics [15]; there is currently no value assigned for the Hybrid procedure. However, we calculated for each patient of the HLHS and variants group A via the comprehensive Aristotle score in order to adjust the complexity according to specific patient and procedural characteristics prior to initial hybrid stage I palliation, as a Norwood procedure would have be performed as the usual standard approach.

**Fig. 1:**
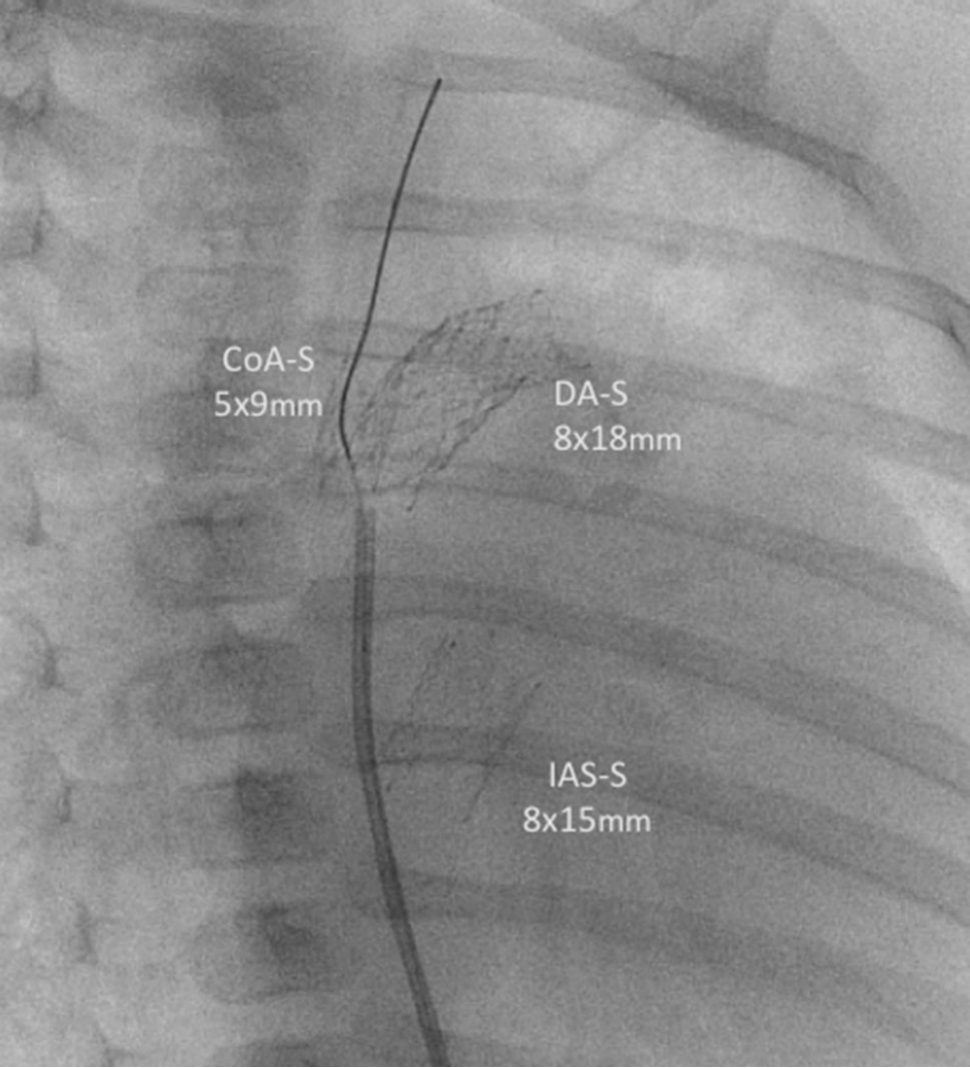
Depicted is a cine picture in right anterior oblique (30°) view with a Sinus-Superflex-DS stent (8 × 18 mm) in the duct, Sinus-Repo-DS stent (5 × 9 mm) within the coarctation, and a Sinus-Superflex-DS (8 × 15 mm) positioned in the atrial septum. A multipurpose 4Fr catheter is still placed in the descending aorta, a coronary 0.014 inch guide-wire advanced through the stent in the coarctation

Risk factors examined for their potential influence on procedure-related mortality included body weight at surgery, morphology of HLHS and variants, presence of aortic atresia, additional cardiac anomalies, and comprehensive Aristotle score. Demographic data including surgical procedures of the patients primarily classified as HLHC are summarized in Table 2. The time and mode of corrective surgery as well as the follow-up survival was analyzed. Operative mortality of all surgical procedures was defined as mortality within 30 days or prior to hospital discharge. Additional end-points that have been investigated were [18] hospital survival after initial Hybrid stage I palliation, interstage I mortality, survival after stage II procedure, interstage II mortality, and follow-up after total cavo-pulmonary connection (stage III, completion of the Fontan circulation). The local institutional ethics review board approved the study, and the need for parental consent was waived. The anatomic diagnosis of HLHS and HLHC was based on two-dimensional echocardiography in all patients, and in addition on magnetic resonance imaging since 2008. Technical aspects of the initial hybrid (off-pump) palliation have been reported previously [18, 23]. In terms of modifications of the circulation, hybrid approach consists of bilateral pulmonary banding (bPAB), percutaneous duct stenting, or individual cases long-term prostaglandin infusion. If required, atrioseptostomy optionally with stent placement was performed. These interventions ensure protection of the lungs from pulmonary hypertension, preservation of an adequate systemic perfusion, and unloading of the left atrium, respectively. Briefly, in elective patients, the hybrid stage I procedure is performed via median sternotomy for branch pulmonary artery banding using 3-mm (bodyweight less than 2.5 kg) or 3.5-mm Gore-Tex (W.L. Gore, Newark, DE) rings [4]. Stenting of the ductus arteriosus was performed in all patients by percutaneous transcatheter approach via femoral vein or by 4F arterial access since the redesigned self-expandable stent (Sinus-superflex-DS) has been available [23]. Heart catheterization for duct stenting and atrial septum manipulation was performed in sedated patients breathing spontaneously, routinely after surgical bPAB. In rescue situations, surgical bPAB was performed in case of a pulmonary run-off caused by a huge interatrial defect, duct stenting to reopen a duct insensitive to prostaglandin-E1, and atrial septal or pulmonary vein manipulation to solve life-threatening pulmonary vein obstruction [21, 23, 24]. Stent size and positioning within the duct was based on a right lateral oblique 30° and 90° lateral view angiography, which were performed by a 4F Judkins or 4F multipurpose catheter positioned either in the pulmonary trunk and/or descending aorta, by hand injection (Fig. 1). Initially, different types of balloon-expandable stents were used. Since 2006, nearly exclusively self-expandable Sinus-Repo 5F and currently Sinus-Superflex-DS stents deliverable through a 4 F sheath with different widths of 7,8,9 and 10 mm and lengths (12, 15, 18, 20 mm) with the CE-mark for duct stenting in newborns (OptiMed Inc. Karlsruhe, Germany) were used. Choice of the stent was largely influenced by the ductal anatomy and the morphology of the ductal-aortic junction. In a subgroup of patients with narrowed aortic isthmus or aortic coarctation, additional short stents (5x9 mm) were placed within the isthmus; currently the Sinus-repo self-expandable stent even with CE-mark for pediatric CoA-stenting is available. Therefore, flow acceleration or narrowing of the color Doppler jet indicating obstructive retrograde coronary flow was not per se considered as a contraindication for a hybrid approach in patients with aortic atresia. The adequacy of a defined atrial septal communication was determined by echocardiographic and in some by invasive hemodynamic measurements. If the atrial septal communication was found to be restrictive, a balloon atrial septostomy or deployment of an atrial septal stent was performed (Fig. 1); in patients, intact atrial septum re-opening was achieved by Brockenbrough technique or by application of high-frequency perforation. In two patients, total anomalous venous return was re-connected to the left atrium by catheter technique [24]. For the last 5 years, the patients were discharged home on medication with bisoprolol and lisinopril in a dosage of 1 × 0.1-(0.2) mg/kg, respectively; digoxin, furosemide, and aspirin are routinely avoided. The standard protocol for outpatient follow-up during the interstage I includes clinical visits in 1-2-week intervals. Patients in the univentricular HLHSgroup were not referred for elective complete invasive hemodynamic and angiographic evaluation prior to hybrid stage II, unless a hemodynamic issue was suspected. However, hemodynamic assessment was performed routinely before completion of the Fontan-circulation. Since 2002, as hybrid stage II was first time described by Akintürk et al. [1], several modifications were introduced and adapted mostly based on the patient’s individual anatomy. In case of an innominate subclavian artery, the segment of it’s arterial wall was included in the aortic arch reconstruction [27]. Aortic arch reconstruction without circulatory arrest has become routine and even the entire comprehensive stage II procedure without cardiac arrest has been possible in several patients [28]. The stage II surgical reconstruction consisted of amalgamation of the proximal ascending aorta with the main pulmonary artery, removal or resection of the stented ductus, aortic arch reconstruction, atrial septectomy (removal of atrial septal stent), removal of the branch pulmonary artery bands with routine angioplasty or left pulmonary artery stenting, and bidirectional cavo-pulmonary connection. Left bidirectional Glenn was performed if a left SVC without a bridging vein was present. Considering a Fontan-circulation, a total cavo-pulmonary connection was performed as a stage III by utilizing an extra-cardiac conduit in all but two patients and in the majority with surgical fenestration (*n* = 40). Transcatheter technique [19] was also used for re-opening or for creation of a new fenestration, if necessary (*n* = 16). Fenestrations were closed in some during the follow-up. The variants of biventricular repair were described previously [2, 18]. The detailed analysis of all patients, who received a biventricular repair after initial “Giessen Hybrid approach” for hypoplastic left heart variants were presented at the AATS 2014, the entire data set is submitted for publication [29].

The surgical technique of HTX with the special aspects of the morphology in patients with HLHS was already reported [7, 13].

### Statistical Analysis

Data are presented as medians (ranges) or means (±standard deviation) and as absolute counts with percentages where appropriate. Continuous variables were compared using a Mann–Whitney test and Student’s *t* test. Kaplan–Meier survival curves using single and cumulative end-points for death were stratified by type of initial palliation. Analyses of risk factors for mortality at different time points for the entire cohort and subgroups, as well as logistic regression were performed.

## Results

**Fig. 2:**
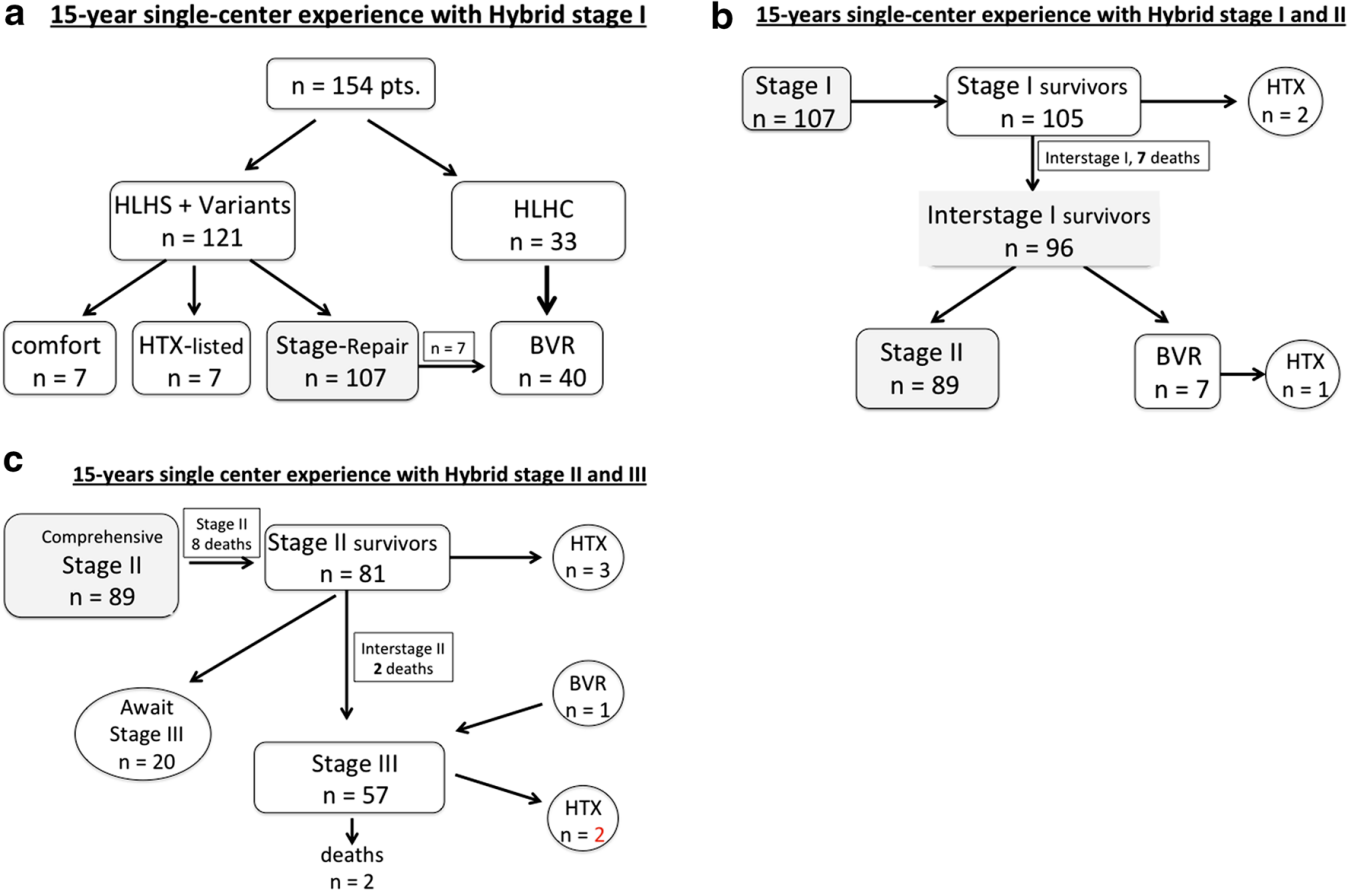
**a**, **b**, **c** shows the flow chart of all patients treated by Hybrid approach. *BVR* biventricular repair, *HLHC* hypoplastic left heart complex, *HLHS* hypoplastic left heart syndrome, *HTX* heart transplantation

**Fig. 3:**
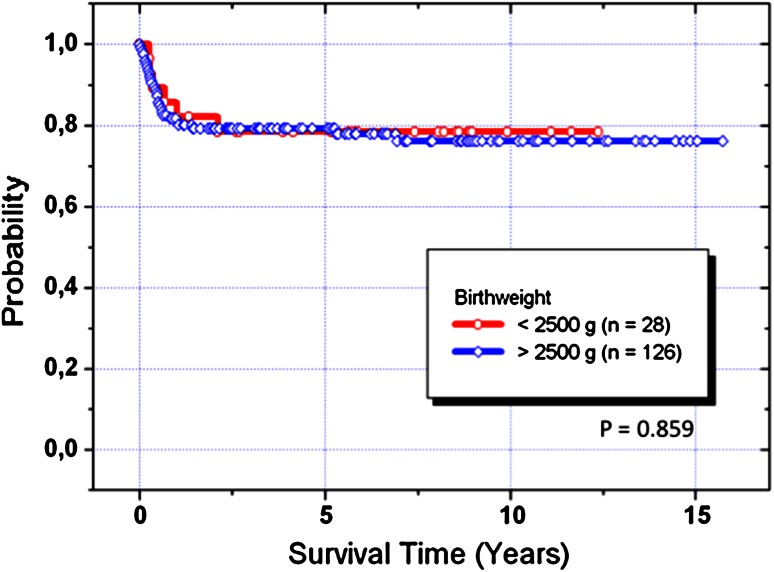
Kaplan–Meier acturial survival curve of all (*n* = 154) patients treated by hybrid approach, differentiated by birth weight less than 2.5 kg

In this longitudinal review the end-point for outcome after initial hybrid approach was analyzed over a time period of 15-years. The analyzed cohort consisted of 154 (57 female) patients (121 HLHS+ variants, 33 HLHC) born with mean body weight of 3.1 ± 0.8 kg; 28 neonates had a weight less than 2.5 kg (minimum 1.21 kg), of those fifteen were categorized to univentricular group A (*n* = 107). The follow-up after Hybrid stage I procedure was complete for all of 120 surviving patients with a median of 5.3 (0.3–15) years. Figure 2a–c summarize the results for each procedural step, with focus on the results of staged palliative approach. In consent with the parents, seven patients were not further treated despite a successful hybrid stage I approach because of chromosomal anomalies or other severe comorbidities. One of them with a complex HLHS variant is still alive at the age of 7 years, only palliated by hybrid stage I. From the remaining 147 patients, seven newborns were primarily listed for HTX and for six of them a donor heart was available; two patients died after HTX. The actual survival of all (*n* = 154) unselected patients treated with hybrid stage I was 80, 78, and 77 % after one-, two-, and fifteen-year, respectively. Birth weight less than 2,500 g did not have an impact on mortality (Fig. 3).

### Hybrid Stage I and Interstage I

**Fig. 4:**
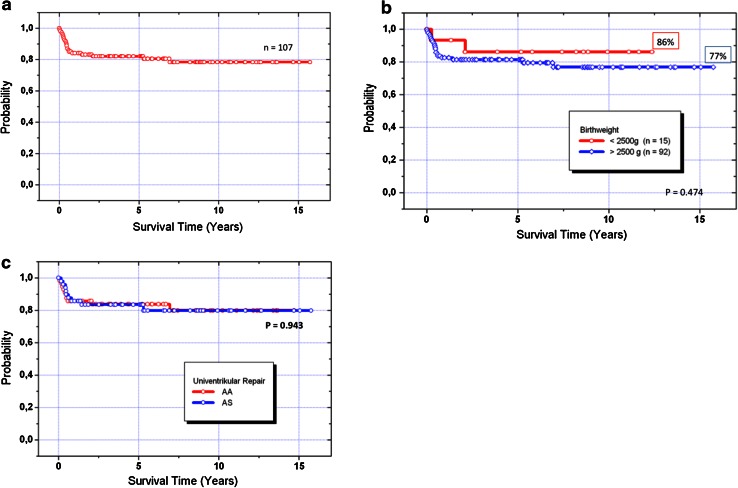
Kaplan–Meier acturial survival curve: **a** HLH-LS(MA/AA/MS/AA, MA/AS, MS/AS + variants): candidates for Norwood I Giessen experience June 1998–June 2013. **b** All patients of group A differentiated by birth weight less than 2.5 kg. **c** Patients of group A (*n* = 107), differentiated in term of aortic atresia (AA, *n* = 58) and stenosis (AS, *n* = 49). *AA* aortic atresia, *AS* aortic stenosis, *MA* mitral atresia, *MS* mitral stenosis

Surgical bilateral PAB was performed at a median age of 7 (range 0–237) days and duct stenting in 151 patients at 10 (2–313) days, respectively. In three patients, ductal patency was achieved by continuous prostaglandin infusion, but in one, secondary duct stenting was performed 6 weeks later. During the initial hybrid procedure, sixteen (11 %) newborns received 2 stents. In 31 patients, manipulation of the interatrial septum (Rashkind) was necessary, either therapeutically in restrictive ASDs or prophylactically especially in a thicker interatrial septum when a compromise of the left atrial drainage could be foreseen. A stent was placed in the atrial septum at the time of stage I in two patients. In 6 patients, a stent was positioned in the aortic isthmus. During the interstage I, repeated duct stenting was necessary in 17 patients, interatrial stenting in 12 patients and isthmus stenting in 4 infants, respectively. No procedural mortality was registered, but five patients did not recover from heart failure despite technically uneventful hybrid approach. Two of them were successfully heart transplanted, and three listed for HTX. Four interstage deaths occurred suddenly at home, in three of them associated with far distance interstage observation. Hospital mortality following initial Hybrid stage I palliation was 1.3 % for the entire cohort. Considering patients with HLHS and variants calculated mortality was 1.8 %. The two hospital deaths, one during surgical pulmonary banding and another one during duct stenting, were already reported previously [18]. However, since 2001, none of the following 134 consecutive patients died during hybrid stage I approach. The modified comprehensive Aristotle score of group A was 18.3 ± 3.0 (mean). Aortic atresia was present in 68 of 121 (56 %) patients, respectively. The end-point analysis concerning cumulative mortality along the sequential steps of the hybrid approach including Stage II and III palliation, presence of Aristotle score of 19 or higher (*p* = 0.267), birth weight less than 2.5 kg (*p* = 0.474), and aortic atresia (*p* = 0.943) was not associated with lower survival (Fig. 4a–c). The overall 15-year actuarial survival for patients with AA was 77 %. Interstage I mortality for group A was 6.7 % (Fig. 2b); none of group B died after hybrid stage I, from group C, one out of seven died on the waiting list for HTX. Additionally, two patients were listed during the interstage I and underwent HTX successfully.

### Follow-Up Data

**Fig. 5:**
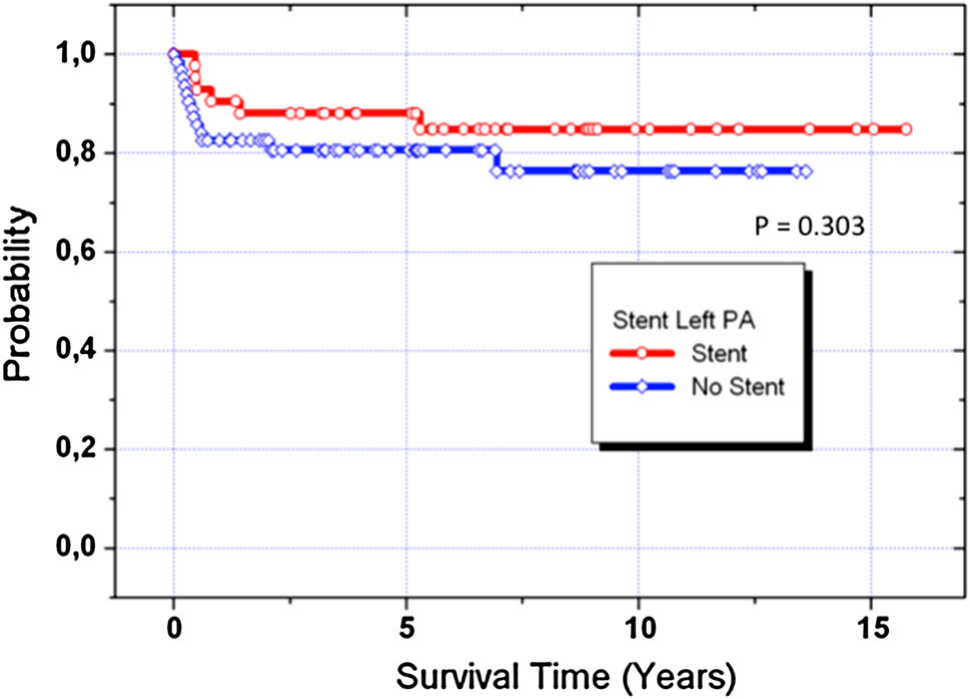
Kaplan–Meier acturial survival curve: Patients of group A (*n* = 107), differentiated in term of left pulmonary artery stenting (LPA, *n* = 38) and stenosis (AS, *n* = 49)

From the non-transplanted 96 interstage I survivors, seven could be switched for biventricular repair, and all of them survived the operation. However, two of them developed a severe post-capillary pulmonary hypertension. One was successfully heart transplanted; the other was referred to Boston for resection of endocardial fibroelastosis with consecutive BVR. However, the latter patient needed finally to be reconverted to a successful TCPC. Biventricular repair was performed in 33 postnatally selected neonates and in 7 patients during follow-up by a cross-over from group A to B, respectively. Median age at BVR was 203 (52–417) days (Table 2). The actuarial survival rate of biventricular repair was 89 %; there were two surgical deaths, and two additional sudden deaths occurred in third and fourth months after BVR at home. Chromosomal or a syndromale disease (*n* = 10) did not influence the BVR mortality. The hospital mortality for comprehensive stage II was 9 % (8 of 89, Fig. 2c); in two of them alternative HTX listing was refused by the parents for religious reasons. ECMO service after comprehensive stage II was necessary in five patients, two of them survived. Following stage II, left pulmonary artery stenting was necessary in 38 % (34 of 89) patients without influencing the mortality (*p* = 0.304; Fig. 5). After interstage II, before completion of the Fontan circulation, three additional patients were successfully heart transplanted; two further patients died and one already listed for HTX. Therefore, transplant-free survival of comprehensive stage II was 85,4 %. Up to now, fifty-seven patients received stage III completion (53 %) and twenty patients (18.6 %) are still waiting for stage III and seven of 107 patients (6.5 %) could be converted into group B. The actuarial survival of all patients of group A after one-, five-, and fifteen years was 84, 82, and 77 %, respectively.

## Discussion

The 15-year single center experience with the hybrid stage I was focused on the outcome of all stages of univentricular palliation and corrective biventricular surgery, respectively. Considering our local setting, the hybrid stage I approach fulfilled all attributes of a highly effective therapeutic tool to treat neonates with duct-dependent systemic blood flow even in patients with a high Aristotle score. Therefore, the retrospective analysis was not intended to compare the “Giessen-Hybrid” with classical Norwood or Sano palliation or differently performed Hybrid procedures. However, 15 years ago, our institutional outcome with the classical Norwood procedure was comparable with outcome data reported by Ashburn et al. [3] in the year of 2003. They reported that only 28 % of neonates undergoing classical Norwood procedure reached a stage III-Fontan completion. Based on our institutional results with the classical Norwood procedure at this point in time and the already published “Hybrid” idea from Gibbs et al. [12], in June 1998, the Hybrid stage I program in Giessen was started. The first patient, who is still living in a good clinical condition, was referred as a newborn in an acute cardiovascular failure because of a postnatal pulmonary run-off. High urgency bPAB saved his life. After clinical stabilization, the duct was stented by a technique, which had been already established to palliate neonates selected for HTX [13]. He was also the first worldwide, who received a successful comprehensive stage II, followed by Fontan completion. Our initial hybrid series was published in 2002 [1]. Based on the promising initial experiences, the hybrid approach developed to our first choice procedure for all newborns with HLHS and variants. Since 2001, bPAB and percutaneous duct stenting were also used for a small group of newborns with HLHC with the goal to avoid neonatal Norwood procedure or to delay a high-risk surgical approach together with expanding surgical options in later infancy without compromising the survival of neonatal patients but still keeping the option for a uni-or biventricular circulation [18]. A demanding controversy, however, exists in the management of this subset of patients with borderline small left heart, e.g. HLHC. In our institution lastly, the decision for hybrid versus “traditional” surgical approach was performed on our surgeon’s risk adjustment [29]. The criteria for cross-over from postnatal univentricular to two ventricle approach 6–8 months later is based on the inflow and outflow characteristics of the grown-up left ventricle obtained by echocardiographic and additional magnetic resonance imaging data. However, the results of BVR remains in some patients questionable, as has been demonstrated in these two patients, in whom the diastolic dysfunction of the left ventricle resulted in unacceptable pulmonary hypertension with consecutive need for HTX and single ventricle re-palliation, respectively. The detailed analysis of all, lastly biventricular-repaired patients and its further discussion are presented elsewhere [29].

The first larger series of Hybrid transcatheter-surgical palliation as a basis for univentricular or biventricular repair was published in 2007 [2]. Until now risk factors as low birth weight, high Aristotle score or aortic atresia did not statistically influence the15-year outcome data. Procedural mortality by the “Giessen Hybrid” stage I approach remained rare. Following the first and until now unique surgical death during hybrid stage I approach, we encounter that meticulous surgery and minimized circulatory compromise by anesthesiological efforts play a key role for successful Hybrid stage I. Therefore, duct stenting and atrial septum manipulations including stenting were performed in the vast majority of patients extubated, only sedated and breathing spontaneously. Innovative self-expandable stents CE-certified for duct and coarctation stenting in neonates with HLHS further optimized the interventional approach [18, 23]. Considering the morphologic variability of the ductal-aortic junction, the high incidence for atrial septum manipulation as well as the occasional presence of an aortic coarctation, we favor the percutaneous approach after surgical bPAB. Meanwhile, the term “Hybrid approach” is used for diverse procedures [9, 10, 14, 26]. Therefore, our outcome analysis should not be generalized to other described hybrid methods. The data of the Pediatric Heart Network report [20] showed a one-year transplant-free survival of 64 versus 74 % for the Norwood and Sano cohorts, respectively. The rate of serious adverse events was up to 46 %, representing a significant morbidity. Initial hybrid treatment, including patients with a high Aristotle score is highly effective in case of prostaglandin resistant duct obstruction, pulmonary run off, or restrictive/intact atrial septum [21, 23, 24]. In addition, the Hybrid stage I approach allows postnatal resuscitation even with the option of later compassionate therapy based on the parent’s decision-making. However, the therapeutic objective is to achieve a long-term prospect for patients, which is worth living after staged procedures finalized by Fontan completion or after biventricular repair. The Achilles’ heel of the hybrid approach was the interstage I period, the fate of the left pulmonary artery after comprehensive stage II, and the diastolic dysfunction in two patients directed from univentricular strategy to BVR. Based on the growing body of literature, that the brain of newborns with HLHS develops with some delay, thereby being more vulnerable to injury from open-heart surgery [16], the authors are convinced, that in principle the hybrid approach allows for a normal neurological outcome, and can stand the comparison with the Norwood palliation. Currently, a prospective study considering this topic is ongoing, and the results of the first almost twenty analyzed patients are promising. However, without considering the Aristotle score at admission, normal somatic and neurological outcome can only be achieved, if there is no negative impact of the surgical-interventional strategy per se. The interstage deaths and also the need for HTX between the initial procedures and even after comprehensive stage II demonstrate these vulnerable periods. The observation of late, post stage II deaths in two , and the need for HTX in additional three patients demonstrate even more that not all surviving patients have the chance for transplant-free outcome. The same is true after Fontan completion and even in patients after BVR. Although the presented data are reporting about 15-year experience with the hybrid approach, the median observation period is still to short to reach a conclusion, which neonates really benefit from the hybrid procedure in terms of the long-term outcome.

### Study Limitations

The retrospective design of this study precluded the assessment of risk factors not entered in the model. Complete data of known risk factors for mortality, such as prenatal diagnosis, were not available for many patients and were not included in the analysis. We also identified a high level of correlation between some of the variables, which may have confounded the multivariable analysis and prevented us from identifying independent associations between variables and the outcome measures. Finally, the duration of follow-up is currently limited to a median of 5.3 years.

## Conclusion

It remains to be seen whether these encouraging results are reflected in improved longer-term outcome for these patients. The ongoing prospective neurological follow-up data collection will tell us if this strategy might have additional beneficial aspects for most patients born with HLHS.
